# Looking at the bigger picture: how the wider health financing context affects the implementation of the Tanzanian Community Health Funds

**DOI:** 10.1093/heapol/czy091

**Published:** 2019-01-25

**Authors:** Sabine Renggli, Iddy Mayumana, Christopher Mshana, Dominick Mboya, Flora Kessy, Fabrizio Tediosi, Constanze Pfeiffer, Ann Aerts, Christian Lengeler

**Affiliations:** 1 Department of Epidemiology and Public Health, Swiss Tropical and Public Health Institute, Socinstrasse 57, Basel, Switzerland; 2 University of Basel, Petersplatz 1, Basel, Switzerland; 3 Department of Health Systems, Impact Evaluation and Policy, Ifakara Health Institute, Plot 463, Kiko Avenue Mikocheni, Dar es Salaam, Tanzania, and; 4 Novartis Foundation, Basel, Switzerland

**Keywords:** Tanzania, community-based health insurance, health financing, health system research, operations research

## Abstract

In Tanzania, the health financing system is extremely fragmented with strategies in place to supplement funds provided from the central level. One of these strategies is the Community Health Fund (CHF), a voluntary health insurance scheme for the informal rural sector. As its implementation has been challenging, we investigated different CHF implementation practices and how these practices and the wider health financing context affect CHF implementation and potentially enrolment. Two councils were purposively selected for this study. Routine data relevant for understanding CHF implementation in the wider health financing context were collected at council and public health facility level. Additionally, an economic costing approach was used to estimate CHF administration cost and analyse its financing sources. Our results showed the importance of considering different CHF implementation practices and the wider health financing context when looking at CHF performance. Exemption policies and healthcare-seeking behaviour influenced negatively the maximum potential enrolment rate of the voluntary CHF scheme. Higher revenues from user fees, user fee policies and fund pooling mechanisms might have furthermore set incentives for care providers to prioritize user fees over CHF revenues. Costing results clearly pointed out the lack of financial sustainability of the CHF. The financial analysis however also showed that thanks to significant contributions from other health financing mechanisms to CHF administration, the CHF could be left with more than 70% of its revenues for financing services. To make the CHF work, major improvements in CHF implementation practices would be needed, but given the wider health financing context and healthcare-seeking behaviours, it is questionable whether such improvements are feasible, scalable and value for money. Thus, our results call for a reconsideration of approaches taken to address the challenges in health financing and demonstrate that the CHF cannot be looked at as a stand-alone system.

## Introduction

**Key Messages**

- When looking at CHF performance, it is important to consider council-specific CHF implementation practices and the wider health financing context.
- Exemption policies and healthcare-seeking behaviour influences negatively the maximum potential enrolment rate of the voluntary CHF scheme
- Higher revenues from user fees, user fee policies and fund pooling mechanisms can set incentives for care providers to prioritize user fees over CHF revenues
- Major improvements in CHF implementation practices would be needed to make the CHF work, but given the wider health financing context and healthcare-seeking behaviours, it is questionable whether such improvements are feasible, scalable and value for money

Following the publication of the World Health Report 2010 and the formulation of the health-related Sustainable Development Goal 3, Universal Health Coverage (UHC) has gained high priority globally (World Health Organization, 2010; Sustainable Development Solution Network, 2015). UHC implies that everyone has access to needed health services of sufficient quality to be effective without incurring financial hardship (World Health Organization, 2010). However, many low- and middle-income countries have been struggling to implement sustainable health financing strategies. A major problem is the informal nature of their economies, which makes revenue collection to fund health systems difficult. Underlying mechanisms of health financing systems also pose challenges (World Health Organization, 2013). The basis to address these challenges lies in the in-depth understanding of the context-specific and often complex designs and implementation practices of existing health financing systems (World Health Organization, 2010, 2013).

**Figure 1.**
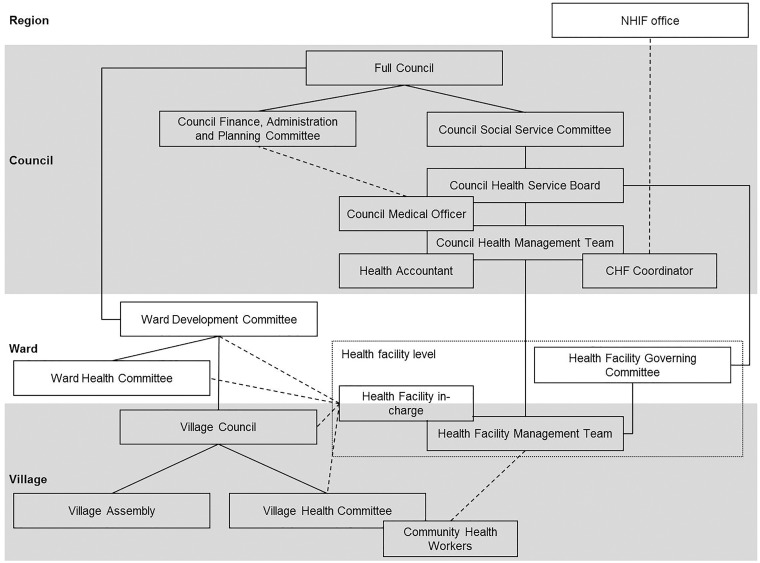
Key stakeholders of cost sharing and insurance funds within a council. Solid lines indicate official reporting hierarchies, dashed lines indicate further relevant interactions and stakeholders within the dotted box belong to the health facility level

In Tanzania, the healthcare system primarily depends on central level funding coming from tax revenues or external donors (Dutta, 2015). There are also several insurance schemes and out-of-pocket payments account for around 23% of total health expenditure (World Health Organization, 2014). Overall, the health financing system is extremely fragmented, both in terms of insurance schemes and within the central level funding system (McIntyre et al., 2008; Haazen, 2012; Borghi et al., 2013; Dutta, 2015). User fees paid out of pocket are levied at the point of access, whereby the councils define the amount to be paid in their user fee policies. National exemption policies stipulate that the poor and other priority groups (children under five, pregnant women, elderly above 60 and people with certain disease conditions, including chronic illnesses, HIV/AIDS, TB and leprosy) are supposed to receive free services at public health facilities (Mubyazi, 2004). All public servants are compulsorily enrolled in the National Health Insurance Fund (NHIF) (McIntyre et al., 2008). Voluntary insurance schemes include the Community Health Funds (CHFs) for the informal rural population (Haazen, 2012). Each council is responsible for administrating its own CHF and defining the benefit package and flat rate premium per year. The CHF scheme covers a whole household. CHF funds raised are doubled through matching grants from the central government via the NHIF (Joseph and Maluka, 2016). Resources collected through CHF revenues, matching grants, user fees and NHIF reimbursements are referred to as ‘Cost Sharing and Insurance Funds (CSIFs)’ (Ifakara Health Institute, 2013). The pooling mechanism of these funds is defined by the councils. Key CSIFs stakeholders within a council are described in Box 1 and Figure 1.

**Box 1. Key stakeholders of Cost Sharing and Insurance Funds within a council (Figure 1)**

Council level

The Council Health Service Board (CHSB), consisting of community and private health sector representatives, is the governance body overseeing the Council Health Management Team (CHMT) (Kessy et al., 2008; Kessy, 2014). The CHSB is responsible for the management and administration of the CSIFs (Mtei and Mulligan, 2007; Kessy et al., 2008). This includes mobilizing and allocating funds, issuing CHF membership cards to exempted households and verifying the collection and expenditure of funds (United Republic of Tanzania, 2001). The CHSB receives technical input from the CHMT through the Council Medical Officer. The CHMT is in charge of monitoring and assuring the quality of services provided (United Republic of Tanzania, 2001). The CHF and NHIF coordinators are typically members or co-opted members of the CHMT (Borghi et al., 2015). The CHF coordinator, who is supported by a council health accountant, oversees the operation of the CHF and tracks membership, fund generation and use (Borghi et al., 2015). It is the duty of the council (often the CHF coordinator) to claim the matching funds from the NHIF. The NHIF coordinator compiles the NHIF claim forms and forwards them to the NHIF office. NHIF reimburses the council or directly the health facility for expenses based on the submitted claim forms.

Ward and village level

The Ward Development Committee (WDC) at ward level and the Village Council (VC) at village level are in charge of sensitizing and mobilizing community members (e.g. during the Village Assembly) and identifying poor households eligible for exemptions (United Republic of Tanzania, 2001).

Health facility level

At facility level the Health Facility Governing Committees (HFGCs), composed of community representatives, oversee the facility operations. They are responsible for the mobilization of financial resources to run the health facility and liaising with the CHSB (Kessy et al., 2008; Kessy, 2014). The Health Facility Management Team (HFMT) enrols community members into the CHF, collects contributions (CHF revenues, user fees) and completes NHIF claim forms (Kessy, 2014; Borghi et al., 2015).

National CHF enrolment rate in 2015 was around 4.5% (Ministry of Health Community Development Gender Elderly and Children et al., 2016), indicating that the target of 30% enrolment by 2015 had not been reached (Ministry of Health and Social Welfare, 2009, 2015). Numerous studies have investigated reasons for low enrolment. Among them are low quality of care, high premium rates, limited benefit packages, lack of trust in the scheme or healthcare provider and failure to see the rationale of an insurance scheme (Kamuzora and Gilson, 2007; Mtei and Mulligan, 2007; Kessy et al., 2008; Stoermer et al., 2011, 2012; Ministry of Health and Social Welfare, 2012; Borghi et al., 2013; Macha et al., 2014; Maluka and Bukagile, 2014; Kalolo et al., 2015, 2018; Kapologwe et al., 2017). Additionally, issues in governance were observed in terms of insufficiently capacitated or functioning CHSBs, HFGCs and WDCs and regarding the role of the NHIF in managing the CHF (Kamuzora and Gilson, 2007; Mtei and Mulligan, 2007; Kessy et al., 2008; Stoermer et al., 2011, 2012; Borghi et al., 2013, 2015; Ministry of Health and Social Welfare, 2013; Kessy, 2014; Maluka and Bukagile, 2014; Mkumbo and Masbayi, 2014; Kalolo et al., 2015, 2018; Joseph and Maluka, 2016). Some studies also described problems of insufficient council management commitment, high administration cost, inadequate supportive supervision, a weak medical supply chain and missing mechanisms for service purchasing, claim processing and risk equalization or cross-subsidization (Kamuzora and Gilson, 2007; Mtei and Mulligan, 2007; Kessy et al., 2008; Stoermer et al., 2011, 2012; Borghi et al., 2013, 2015; Macha et al., 2014; Maluka and Bukagile, 2014; Joseph and Maluka, 2016). Furthermore, inadequate fund pooling, insufficient transparency and accountability, as well as poor data quality and management were mentioned in connection with low CHF enrolment (Kamuzora and Gilson, 2007; Kessy et al., 2008; Stoermer et al., 2011, 2012; Ministry of Health and Social Welfare, 2012; Borghi et al., 2013, 2015; Frumence et al., 2014; Macha et al., 2014; Maluka and Bukagile, 2014; Mkumbo and Masbayi, 2014; Kalolo et al., 2015, 2018; Joseph and Maluka, 2016). Lastly, exemption policies were reported to potentially discourage people from joining the CHF (Kamuzora and Gilson, 2007; Mtei and Mulligan, 2007; Kessy et al., 2008; Nangawe, 2012; Idd et al., 2013; Maluka, 2013; Ministry of Health and Social Welfare, 2013; Macha et al., 2014).

However, little detailed evidence has been provided about how CHF implementation is affected by council-specific CHF implementation decisions. These council-specific implementation practices, which differ from one council to the other, include the overall CHF administration and the definition of the premium and benefit package. Neither is there much information about how the success of these council-specific CHF implementation practices is influenced by the wider health financing context, meaning council defined user fee policies and fund pooling mechanisms as well as exemption policies and other health financing mechanisms. Hence, this article aims to investigate council-specific CHF implementation practices and how these practices and the wider health financing context within a council affect CHF implementation and therewith potentially enrolment.

## Methods

### Description of study councils

Two rural councils ‘A’ and ‘B’ from the same region were selected. Both benefited from the ‘Initiative to Strengthen Affordability and Quality of Healthcare (ISAQH)’, with which the authors were associated and which aimed to expand CHF coverage through: (1) CHF implementation training for all relevant stakeholders (2012), (2) CHF forum (2013), (3) CHF radio spots (2012–14), (4) supportive supervision on CHF data management (2012–14) and [5] village sensitization meetings (2012 for both councils and 2013 for council A only). Councils were chosen because of their difference in perceived CHF implementation capacity as judged by ISAQH staff. Council A was perceived as better performing than council B. Relevant council characteristics and specific health financing decisions (CHF premium, CHF benefit package, user fee policies and fund pooling mechanisms) are described in Table 1. Supplementary Figure S1 summarizes CHF administration activities reported to be conducted by each council.

**Table 1.** Description of study councils (status 2014)

Characteristics	Council A	Council B
Population size^a^	∼250 000	∼400 000
Average household size^a^	4.9	4.3
Number of health facilities^b^	38	59
Number of public health facilities (hospitals/health centres/ dispensaries)^b^	27 (23/3/1)	25 (20/5/0^g^)
Perceived CHF implementation capacity	Medium	Low
Year of CHF introduction^c^	2003	2008/9
CHF premium^c^	3.01/6.02 USD^e^^,f^	6.02 USD^f^
CHF benefit package^c^^,d^	Maximum of six beneficiaries from one household per CHF card and unlimited access to all services offered at any public health facility within the council, including the council hospital	Maximum of five beneficiaries from one household per CHF card with access limited to all services offered at the health facility, where CHF registration took place
User fee policy^d^	‘Fixed’ (independent of treatment): 0.90 USD at public dispensaries or health centres including all services; 1.20 USD at the public hospital for registration/consultation and various prices for medical supplies, diagnostics or any other additional services	‘Flexible’ (depending on treatment): 0.12–1.08 USD for registration/consultation and various prices for medical supplies, diagnostics or any other additional services at all public health facilities
Fund pooling^d^	Cost Sharing and Insurance Funds pooled at council level	Cost Sharing and Insurance Funds pooled at health facility level
Role of CHF coordinator	Dental Medical Officer at council hospital	Health facility in-charge (medical officer) at main council health centre

^a^ National Bureau of Statistics (2013).

^b^ *Source:* Comprehensive Council Health Plans of selected councils collected by SR and IM.

^c^ *Source:* CHF reports of selected councils collected by SR and IM.

^d^ *Source:* Informal personal communication and observational data from selected councils collected by SR and IM.

^e^CHF premium changed from 3.01 USD to 6.02 USD mid-October 2014.

^f^Annual average exchange rate for 2014 (1662 TSh = 1 USD) (Bank of Tanzania, 2017).

^g^There is a designated non-public referral hospital in council B.

### Routine data collection

Routine data relevant for the understanding of council-specific CHF implementation practices (overall CHF administration and definition of premium and benefit package) and the wider health financing context (user fee policies, fund pooling mechanisms, exemption policies and other health financing mechanisms) were collected at public health facility and council level for the financial year (FY) 2013/14 or the calendar year 2014 between February and March 2015.

#### Data collected at public health facilities

We collected data on the number of households enrolled in the CHF, the number of out-patient visits by financing source (CHF, NHIF, exempted, user fee), as well as the amount of revenues by financing source (CHF, user fee, other) and expenditures from all public health facility for each month in 2014. In council B, one dispensary could not be reached due to its remote location.

Yearly averages for CHF enrolment, the number of out-patient visits, revenues and expenditures by health facility level (dispensary, health centre, hospital) were calculated for 2014 (if not specified otherwise). Total council figures were based on health facility level averages and the total number of public health facilities per council, except where indicated otherwise. Revenues and expenditure were converted from Tanzanian Shillings (TSh) to USD using the annual average exchange rate for 2014 (1662 TSh = 1 USD) (Bank of Tanzania, 2017).

The required routine data were often available owing to a data collection sheet distributed to all public health facilities by ISAQH. To cross verify the data and fill gaps, other available documentation was used. This included CHF counter books, CHF register books designed by NHIF, CHF membership cards, CHF receipt books, out-patient registers, monthly or yearly out-patient or financial health facility reports and cash books. In rare cases in council A where no other data source was available reports from the CHF coordinator or ISAQH were used to obtain CHF enrolment data. If data for a particular month could not be found in any of the sources, the average of available months was taken to compute the missing data. In case this could not reliably be estimated, the health facility was excluded from average calculations for that particular value, leading to different numbers of units considered (*N*) in Table 2.

**Table 2:** Routine data collected at public health facilities for the year 2014 by level of care and for the total council

	**Council A**							**Council B**				
	Dispensary (*N* = 23)		Health centre (*N* = 3)		Hospital (*N* = 1)		Total council	Dispensary (*N* = 20)		Health centre (*N* = 5)		Total council
		*N*		*N*		*N*			*N*		*N*	
**Yearly CHF enrolment**												
Households	146	23	328	3	975	1	5327	19	19	97	5	866
**Yearly number of out-patient visits at public health facilities by financing source**												
Total	5946	16	19 458^a^	1	12 821^a^	1	207 951	4127	19	15 115	4	158 108
CHF (% of total)	3202 (54%)	16	6908 (36%)^a^	1	3398 (27%)^a^	1	97 760 (47%)	347 (8%)	2	NA	0	NA
NHIF (% of total)	87 (1%)	16	272 (1%)^a^	1	1018 (8%)^a^	1	3829 (2%)	64 (2%)	2	NA	0	NA
User fee (% of total)	151 (3%)	16	1630 (8%)^a^	1	7831 (61%)^a^	1	16 203 (8%)	1325 (32%)	19	6522 (43%)	4	59 103 (37%)
Exempted (% of total)	2506 (42%)	16	10 648 (55%)^a^	1	574 (4%)^a^	1	90 158 (43%)	2390 (58%)	2	NA	0	NA
**Yearly revenues and expenditure at public health facilities in USD by financing source**												
Total revenue	694	18	2303	2	NA	0	22 881^b^	3008	19	22 125	5	170 781
CHF (% of total)	546 (79%)	18	845 (37%)	2	NA	0	15 094 (66%)b	114 (4%)	19	589 (3%)	5	5225 (3%)
User fee (% of total)	142 (20%)	18	1458 (63%)	2	NA	0	7633 (33%)b	2865 (95%)	19	19 337 (87%)^c^	5	153 982 (90%)
Other (% of total)	7 (1%)	18	0 (0%)	1	NA	0	154 (1%)^b^	29 (1%)	19	2199 (10%)	5	11 575 (7%)
Total expenditure	11	18	193	2	NA	0	834^b^	2619	19	14 167	4	123 222
% spent	2%	18	8%	2	NA	0	4%^b^	87%	19	87%	4	87%

^a^Estimations were based on average data from 2013 as no data for 2014 was available, but this was considered as realistic because CHF enrolment rate at the particular health centre only changed by 0.3% and at the hospital by 6%.

^b^Total council figures do not include the hospital due to unavailability of data.

^c^Includes also user fees collected for in-patient services as this amount could not clearly be separated from the total revenues documented in the health facility.

#### Data collected at council level

At council level, Comprehensive Council Health Plans (CCHPs) and annual combined Technical and Financial Performance Implementation Reports (TFPIRs) were used to analyse the contribution of various funding sources to overall health financing in the FY2013/14. Except for the central government’s in-kind contributions through the Medical Store Department (MSD), funds outside council accounts (contributions from multi- and bilateral partners) were excluded as they could not reliably be tracked within the council system (Ministry of Health and Social Welfare and Prime Minister’s Office Regional Administration and Local Government, 2011). Yet, for reference the contributions from multi- and bilateral partners in council A and B were budgeted to be 1 741 395 USD and 2 338 951 USD in the FY2013/14. In council A, receipts of money submitted by health facility in charges and monthly revenue reports from cash books were obtained from the health accountant. In council B, no such detailed documentation could be obtained. TSh were converted to USD using the annual average exchange rate for the FY2013/2014 (1626 TSh = 1 USD) (Bank of Tanzania, 2017).

### Cost of CHF administration and its financing sources

To explore CHF administration, which is handled independently by each council, we investigated the cost of CHF administration and how the wider health financing context, in particular other financing sources, contributes to this cost. Therefore, an approach similar to the methodology used previously for the CHF in Tanzania was adopted (Borghi et al., 2015). Yearly recurrent costs required for administrating the CHF at health facility and council level were estimated for 2014. For this an ingredient approach was used, whereby quantities of each resource were identified, and valued with the appropriate unit cost (Drummond et al., 2005).

Costs were classified by resource (personnel, per diem, transport, other expenses), financing sources (CHF, NHIF, user fee, other public health financing sources, other public or non-public sources), cost type (variable, fixed) and activity (mobilization, fund pooling, stewardship, purchasing). For categorizing activities, the framework of Mathauer and Nicolle (2011) was used. Personnel cost was defined as the cost of staff time and estimated based on their salary and time spent. When estimating the time spent on activities that were not solely conducted to administer the CHF (e.g. HFGC meetings), costs were apportioned accordingly based on information given by respondents (e.g. proportion of time spent on CHF-related issues) (Supplementary Table S1).

To identify activities, time spent, resources required and financing sources, a pre-defined data collection template was used to interview 22 informants: CHF coordinator, health accountant, Council Medical Officer and one responsible person for CHF administration at six public dispensaries and two public health centres per council. However, in council B only at one of the three visited health centres an informant was available and willing to provide the required information. This resulted in 11 informants in council A and 10 in council B.

Details on cost calculations can be found in Supplementary Annex S1. Overall, council cost was computed by multiplying the average health facility cost with the number of public health facilities per council and adding the council level cost. All costs were calculated in TSh and converted to USD using the annual exchange rate for 2014.

## Results

### Routine data collected at public health facilities

**Figure 2.**
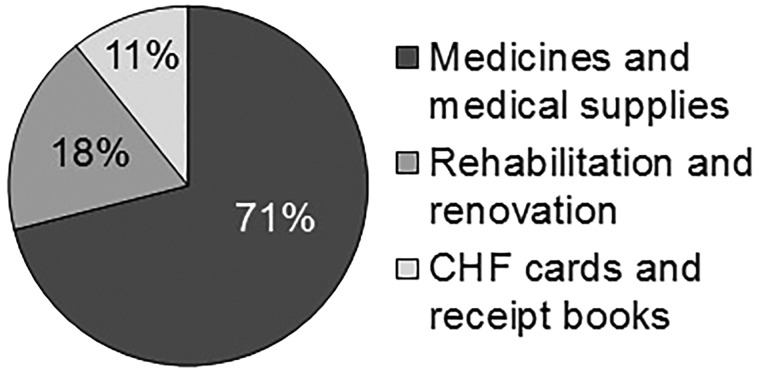
Spending pattern of CHF revenues in council A for the FY2013/14. In council B, no such detailed documentation could be obtained

Table 2 displays routine data collected at public health facilities relevant for understanding CHF implementation in the wider health financing context. CHF population coverage in 2014 was 11.0% in council A and 1% in council B. Strikingly, in council A most out-patients were either exempted or CHF members and only few paid user fees. This was different in council B, where patients were either exempted or paid user fees. Consequently, a big share of revenues collected at public health facilities in council A came from CHF contributions, while in council B the main source of revenues was user fees. Council B had more than seven times higher total revenues. This was primarily due to the greater number of patients paying user fees and the flexible user fee amount, but also because of a smaller CHF benefit package and bigger CHF premium (Table 1). Council A is therefore losing out financially as a result of higher CHF coverage, a smaller CHF premium, a bigger CHF benefit package and fixed user fees (Table 1). The percentage of revenues spent at public health facilities in council A reflected the fund pooling mechanisms in place (Table 1), with a single council level fund pool (account), where only little cash was transferred back to the health facilities for rehabilitation and renovation (Figure 2). In contrast, the proportion of collected money spent was much higher in council B, with individual health facility level fund pools (accounts). Generally, observations across health facilities revealed that reporting formats were inconsistent, patient registers did not capture the financing source of out-patients (CHF, NHIF, exempted, user fee) and in places with more than one person consulting patients CSIFs data was not consolidated.

### Routine data collected at council level

To further understand CHF implementation in the wider health financing context, Table 3 shows the contribution of various funding sources to overall health financing in the two study councils for the FY2013/14 based on routine data collected at council level. Funds are divided into funds approved, brought forward, received and spent. Funds brought forward are unspent funds from the previous year (FY2012/13). CHF revenues only made up around 2% of total funds available for health (sum of brought forward and received). The proportion of CHF money brought forward was high compared with its share in the funds approved, received and spent. This reflected the greater difficulty to spend this money relative to funds from other sources. Council A had less problems receiving (81% of approved budget) and spending (41% of brought forward and received) CHF money in comparison to council B (0.3% received of approved budget and 0% spent of brought forward and received). In contrast to the CHF revenues, revenues coming from other CSIFs were spent easier in both councils.

**Table 3.** Contribution of various funding sources to overall health financing by resources approved, brought forward, received and spent for each council in the FY2013/14

[USD] (% of total)	**Council A**				**Council B**			
	Approved budget	Brought forward	Received	Spent	Approved budget	Brought forward	Received	Spent
Personal emolument (LGBG^a^)	1 421 846 (61%)	0	892 258 (57%)	892 258 (49%)	1 593 944 (49%)	0	1 571 962 (57%)	1 571 962 (59%)
Other charges (LGBG^a^)	119 741 (5%)	18 132 (3%)	130 044 (8%)	103 012 (6%)	221 997 (7%)	0	188 932 (7%)	148 005 (6%)
Health Sector Basket Fund	318 478 (14%)	137 892 (26%)	318 478 (20%)	369 029 (20%)	492 600 (15%)	263 348 (51%)	492 600 (18%)	474 540 (18%)
Health Sector Development Grant	74 124 (3%)	105 677 (20%)	23 067 (1%)	90 023 (5%)	113 809 (4%)	173 893 (34%)	0	164 399 (6%)
Local Government Development Grant	116 875 (5%)	241 604 (45%)	12 303 (1%)	203 936 (11%)	0	14 749 (3%)	0	0
Central government other source	0	0	0	0	246 052 (8%)	0	246 052 (9%)	37 587 (1%)
Council own source	12 303 (1%)	0	0	0	123 026 (4%)	0	0	0
Receipt in kind (Medical Store Department)	167 780 (7%)	0	113 628 (7%)	113 628 (6%)	223 538 (7%)	0	223 538 (8%)	223 538 (8%)
**Cost sharing and insurance funds**								
National Health Insurance Fund	19 721 (1%)	0	9421 (1%)	9421 (1%)	24 605 (1%)	10 102 (2%)	0	10 102 (0%)
Community Health Fund	44 412 (2%)	21 986 (4%)	36 131^c^ (2%)	23 795 (1%)	169 530 (5%)	55 060 (11%)	554(0%)	0
User fee	23 873 (1%)	0	19 242 (1%)	13 274 (1%)	14 563 (0%)	0	14 563 (1%)	14 563 (1%)
Drug Revolving Fund^b^	7382 (0%)	12 841 (2%)	12 215 (1%)	19 944 (1%)	0	0	0	0
**Total**	**2 326 535**	**538 131**	**1 566 786**	**1 838 319**	**3 223 664**	**517 152**	**2 738 200**	**2 644 696**

^a^Local Government Block Grants (LGBGs) are divided into ‘Personal emolument’ (salaries) and ‘Other charges’ (statutory employment benefits).

^b^Money obtained from selling medicines at hospital level (only in councils with a public hospital) (McIntyre et al., 2008).

^c^Composition of CHF (45%) and matching fund (34%) contributions from all levels of care as well as NHIF (14%) and user fees (6%) from health centres and dispensaries. A total of 2% are of unknown source.

Finally, the spending pattern of CHF revenues from council A (23 795 USD) revealed that the revenues were spent as stipulated in the guidelines with at least 70% of expenditure on medicines and supplies (Figure 2).

### Cost of CHF administration and its financing sources

**Figure 3.**
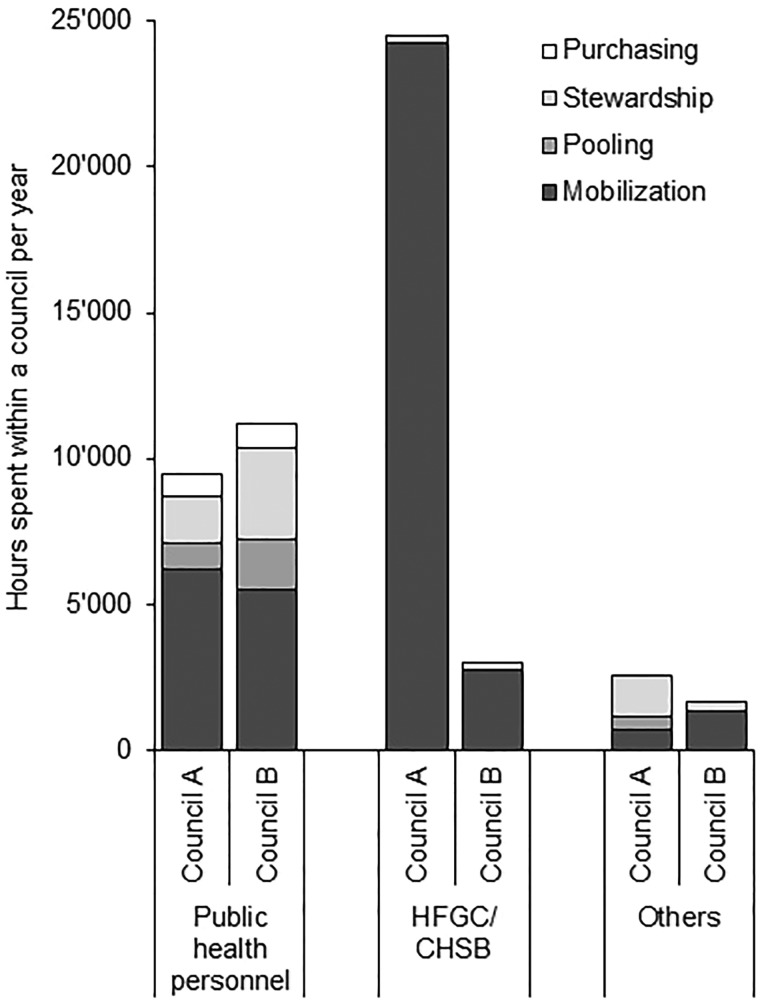
Estimated annual number of hours spent on CHF administration within a council by type of personnel and activity in 2014

Table 4 shows personnel costs (based on salary and time spent) and financial costs (per diem, transport and other expenses) for CHF administration in the councils A and B. In both councils financial costs only made up about 15% of total cost. Mobilizing people to join the CHF (including enrolment) was the most resource-intense activity at health facility level, both in terms of financial and overall cost. At council level, stewardship of the CHF scheme caused the biggest overall cost, but mobilization activities remained with the largest share of financial cost. Fund pooling and purchasing only marginally contributed to the total cost because little time was spent on these activities (Figure 3). In both councils, important drivers for financial cost were CHF supplies (cards, receipt books), transport cost for fund pooling and per diem cost for mobilization, fund pooling and stewardship. Financial as well as overall cost for administrating the CHF was about double in council A compared with council B.

**Table 4.** Average annual health facility level, council level and council overall cost in USD by input, council, type of resource and activity^a^ for 2014

	**Council A**						**Council B**					
	Personnel	Per diem	Transport	Other expenses^b^	**Total financial** ^c^	**Total overall** ^d^	Personnel	Per diem	Transport	Other expenses^b^	**Total financial** ^c^	**Total overall** ^d^
**Dispensary level**												
Mobilization	2735	0	0	127	***127 (4%)***	***2861 (87%)***	753	68	0	18	***86 (10%)***	***839 (63%)***
Fund pooling	103	0	68	0	***68 (40%)***	***171 (5%)***	197	0	68	0	***68 (26%)***	***265 (20%)***
Stewardship	134	86	30	0	***116 (46%)***	***250 (8%)***	160	11	65	0	***75 (32%)***	***235 (18%)***
***Total***	***2971***	***86***	***98***	***127***	***310 (9%)***	***3282***	***1110***	***79***	***133***	***18***	***229 (17%)***	***1340***
**Health Centre level**												
Mobilization	1296	337	0	282	***619 (32%)***	***1915 (76%)***	1776	159	0	85	***244 (12%)***	***2019 (79%)***
Fund pooling	107	0	68	0	***68 (39%)***	***175 (7%)***	6	0	0	0	***0.4 (5%)***	***7 (0%)***
Stewardship	301	55	60	0	***115 (28%)***	***416 (17%)***	399	12	108	0	***120 (23%)***	***519 (20%)***
***Total***	***1703***	***392***	***128***	***282***	***802 (32%)***	***2505***	***2181***	***171***	***108***	***85***	***364 (14%)***	***2545***
**Hospital level**												
Mobilization	3613	193	0	837	***1029 (22%)***	***4642 (81%)***						
Fund pooling	154	0	68	0	***68 (31%)***	***222 (4%)***						
Stewardship	496	245	67	39	***351 (41%)***	***847 (15%)***						
***Total***	***4263***	***438***	***135***	***875***	***1448 (25%)***	***5712***						
**Council level**												
Mobilization	4288	2396	752	0	***3148 (42%)***	***7435 (28%)***	1823	1745	376	0	***2121 (54%)***	***3944 (17%)***
Fund pooling	1100	1092	215	7	***1314 (54%)***	***2414 (9%)***	2215	0	0	2	***2 (0%)***	***2217 (9%)***
Stewardship	10 238	2396	44	581	***3022 (23%)***	***13 260 (49%)***	9913	892	78	52	***1021 (9%)***	***10 935 (47%)***
Purchasing	3723	0	0	2	***2 (0%)***	***3725 (14%)***	6367	0	0	2	***2 (0%)***	***6369 (27%)***
***Total***	***19 350***	***5884***	***1011***	***590***	***7485 (28%)***	***26 835***	***20 318***	***2637***	***454***	***56***	***3147 (13%)***	***23 465***
**Overall council**												
Mobilization	74 687	3599	752	4597	***8949 (11%)***	***83 635 (72%)***	25 758	3904	376	781	***5061 (16%)***	***30 819 (49%)***
Fund pooling	3945	1092	2043	7	***3142 (44%)***	***7087 (6%)***	6192	0	1364	4	***1368 (18%)***	***7560 (12%)***
Stewardship	14 710	4783	984	620	***6387 (30%)***	***21 097 (18%)***	15 107	1163	1911	52	***3126 (17%)***	***18 233 (29%)***
Purchasing	3723	0	0	2	***2 (0%)***	***3725 (3%)***	6367	0	0	2	***2 (0%)***	***6369 (10%)***
***Total***	***97 065***	***9474***	***3779***	***5226***	***18 479 (16%)***	***115 545***	***53 424***	***5067***	***3651***	***839***	***9557 (15%)***	***62 981***

^a^Activities were categorized according to Mathauer and Nicolle (2011).

^b^Others included supplies (e.g. CHF cards and receipts, registration books, printouts) as well as rent, food and refreshment during meetings if applicable.

^c^Values in brackets indicate the percentage of total overall cost for the specific activity.

^d^Values in brackets indicate the percentage of total overall cost for the specific health system level (dispensary, health centre, council or overall council).

Similar to the overall cost, time spent administrating the CHF in council A was more than double the amount of council B (Figure 3). It was however interesting that the number of hours spent by public health personnel in council A was less than in council B. This was mainly because in council A front-line workers at health facility level spent less time on CHF administration (particularly mobilization) than in council B (7% and 25% of a single full-time person at dispensary and health centre level in council A vs 12% and 33% in council B; data not shown) and a large share of this work was taken over by HFGC members.

**Figure 4.**
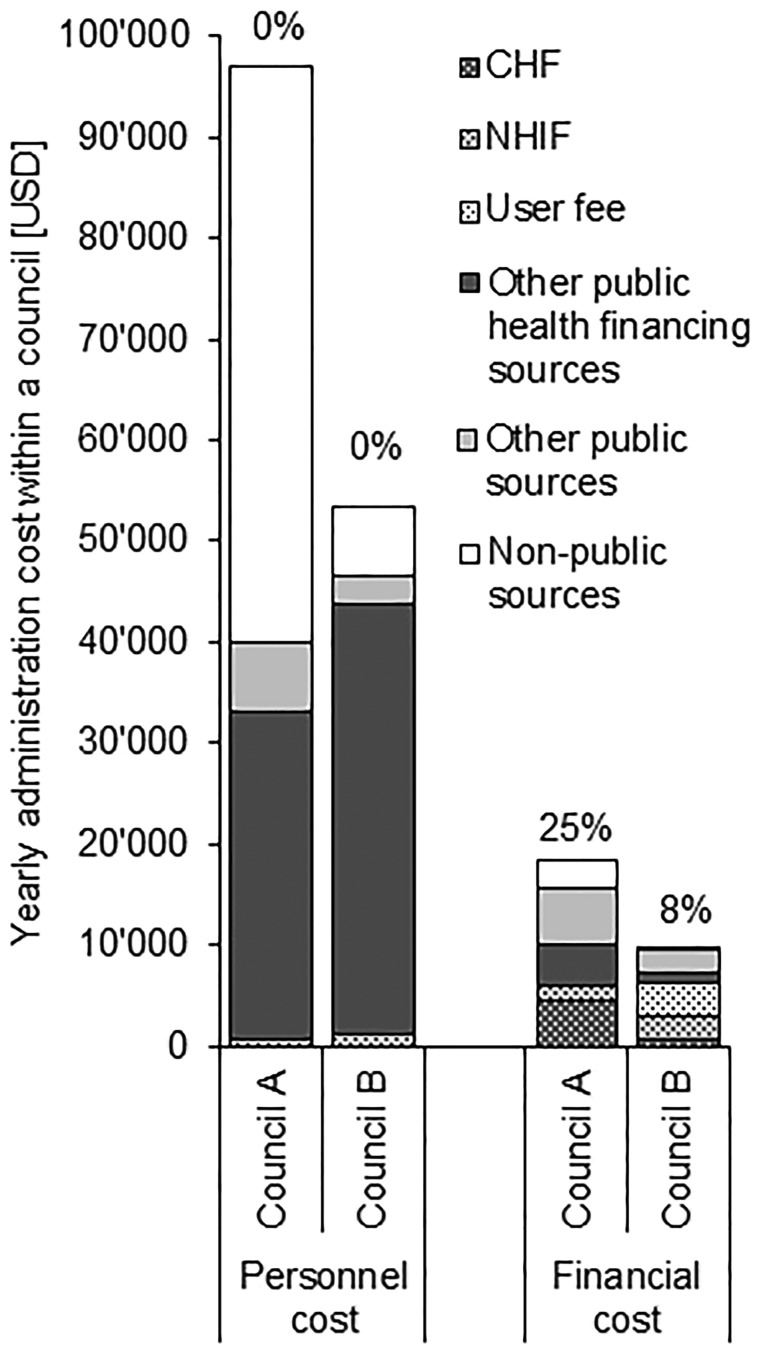
Contribution of different financing sources to personnel and financial cost incurred for CHF administration by council in 2014. Percentage figures indicate the proportion financed by CHF revenues

As a consequence of responsibilities being more equally shared amongst stakeholders in council A (especially with those outside the public sector), personnel costs in council A were financed to a large extent by non-public money (Figure 4). In contrary, in council B personnel costs were mainly carried by the public sector as most of the activities were implemented by public employees. Personnel costs in both councils were exclusively financed through non-CHF money.

Remarkably, only 25% and 8% of the total financial cost for CHF administration were directly financed by CHF revenues in council A and B, respectively. The percentage in council A was higher because these financial costs (CHF cards and receipt books) were pure variable cost and depended on the number of CHF member households. All additional financial costs for CHF administration were borne by other financing sources, including contributions from NHIF and user fees.

In both councils, overall costs mainly consisted out of fixed cost (data not shown). As a result, the administration cost per CHF member household was lower in council A than in council B (Table 5), although overall administration cost was bigger (Table 4). The cost–revenue ratio was 0.50 and 0.92 in councils A and B when only the financial costs were considered. This means the financial administration cost was below the premium paid by a CHF household. When the cost of personnel time was included, the ratio increased to around 3 in council A and 6 in council B, meaning administration cost was more than three or six times above the premium paid by a CHF household. If only considering the administrative cost directly financed through CHF revenues, the cost revenue ratio decreased to 0.12 in council A and 0.07 in council B. This ratio was smaller in council B because administration cost directly financed through CHF money was the same for each household in either council, but premiums were higher in council B. Most importantly, this meant that there was >70% of the CHF revenue left to purchase medicines and supplies and do minor facility renovations (cost paid by CHF revenues/total revenues < 0.3).

**Table 5.** Summary of cost revenue ratios and cost per CHF member household for the year 2014

	Council A	Council B
**Enrolment**		
Total number of individuals enrolled (%)	29 048 (11%)	4186 (1%)
Total number of households enrolled	5327	866
Premium paid by each household [USD]	3.46	6.02
Total revenues (including matching fund) [USD]	18 408 (36 816)	5212 (10 423)
**Administration cost [USD]**		
Cost paid by CHF revenues	4565	742
Financial cost	18 479	9557
Total overall cost (including personnel)	115 545	62 981
**Cost revenue ratio (including matching fund)**		
Cost paid by CHF revenues/total revenues	0.25 (0.12)	0.14 (0.07)
Financial cost/total revenues	1.00 (0.50)	1.83 (0.92)
Total overall cost/total revenues	6.28 (3.14)	12.08 (6.04)
**Cost per CHF member household [USD]**		
Cost paid by CHF revenues/household	0.86	0.86
Financial cost/household	3.47	11.03
Total overall cost/household	21.69	72.72

## Discussion

Strikingly, although population coverage in council A was just above 10%, only few patients at dispensary and health centre level paid user fees. This clearly indicated that the people seeking public care the most were the exempted and insured. The others were either seeking care in the non-public sector, not at all or only at very late stages, when they had to attend hospital level services (as indicated by a high proportion of user fee patients for the hospital). This suggested and confirmed previous findings that CHF enrolment was likely to be affected by healthcare-seeking behaviour and exemption policies, which stipulate free service provision to groups with a higher likelihood to be in need of care (Mtei and Mulligan, 2007; Macha et al., 2014). These factors also undoubtedly influence negatively the maximum potential enrolment rate which could possibly be reached with a voluntary scheme.

On the other hand, the number of patients paying user fees and the council-specific user fee policy, CHF premium and benefit package seemed to impact the total revenues collected. Compared with council B, council A was losing out financially as a result of high CHF coverage (low number of patients paying user fees), fixed user fees independent of the treatment received, a small CHF premium and a bigger CHF benefit package. In contrast, council B had substantial higher revenues due to lower CHF coverage (greater number of patients paying user fees), flexible user fee amounts depending on the treatment received, a smaller CHF benefit package and a bigger CHF premium.

Furthermore, the fund pooling mechanism in place had an influence on the availability of money and the subsequent spending pattern at health facility level. This meant that higher revenues from user fees, a flexible user fee policy and fund pooling at health facility level might have set incentives for the supply side to prioritize user fees over CHF revenues, which also poses a problem for equity. Thus, the situation in council B, where revenues from flexible user fees were high and funds were pooled at health facilities, might have provided little incentives for healthcare workers and HFGC members to conduct CHF mobilization activities. At the same time, the higher CHF premium and a smaller benefit package in council B might neither have provided incentives for the demand side to join the CHF despite the high user fees, which is in contrary to expectations (Kessy et al., 2008). This altogether would contribute to explain why enrolment rate was so low in council B.

Additionally, the decision in council B to pool and use the CSIFs at health facility level led to insufficient documentation at council level. This made it impossible for the council to know what CSIFs were received at health facility level and how they were spent. Neither did it allow applying for matching funds. Fund pooling at health facility level also made it more difficult to put a mechanism in place for balancing the risk across the many smaller pools, which emerged as a consequence. Documentation was about to be improved at the time when the study was conducted, but without addressing the problem of matching fund application or risk pooling. The latter problems were also reported from other councils elsewhere in the country, whereby the fragmented risk pools were seen as a challenge to equity (Borghi et al., 2013). In contrast, pooling of CSIFs at council level in council A facilitated planning and budgeting as well as risk pooling and other CHF administration processes. This was observed based on the bigger percentage of budgeted CHF revenue received and available revenues spent as well as due to the possibility to request for matching funds, track how available revenues were used and allow for risk sharing through need-based reallocation of funds.

Both councils were facing difficulties to spend CHF revenue, because of lengthy and cumbersome overall CHF administration processes attached to it. For example, in council B CHF money collected at council level (prior to the implementation of individual health facility fund pooling) was stuck in the council account and could not be spent because of not clearly defined processes. In council A, use of funds was impeded by the closure of the CHF account and its consolidation with other council accounts, which changed fund access rights. Similar problems with fund usage have been reported by others (Mubyazi et al., 2006; Mtei and Mulligan, 2007; Kessy et al., 2008; Ministry of Health and Social Welfare, 2012; Borghi et al., 2013; Ministry of Health and Social Welfare, 2013; Macha et al., 2014). Also, not knowing the number of CHF patients treated at each health facility from routine data impeded in either of the two councils the possibility of risk-adjusted reallocation of the CHF money.

Overall, these administrative hurdles had an impact on the quality of data available for planning and budgeting and made activities planned to be implemented through CHF revenue more unlikely to happen. The problems of CHF administration additionally led to a financial loss as matching funds could not be requested due to the lack of household registration details and/or proof of money submission, similar to what had been noted earlier (Borghi et al., 2013; Kalolo et al., 2015). Consequently, all these bottlenecks in administration led to CHF implementation failures and therewith diminished potential positive effects of a council level health insurance scheme. This may ultimately also have contributed to CHF member dissatisfaction and low enrolment.

The selection of the same study approach as used previously by Borghi et al. (2015) for assessing the cost of CHF administration allowed for comparison across studies. Importantly, several key findings could be confirmed: (1) lack of financial sustainability of the CHF as such, (2) substantial personnel cost with a share of around 85% of total cost, (3) workload of front-line health workers in a very similar percentage range of a single full-time person, (4) mobilization as the most significant task at health facility level and CHF stewardship at council level, (5) similar relative cost of different administration activities at health facility, (6) comparable average annual health facility level cost for an average dispensary in council B and (7) higher cost per CHF member household in area where enrolment was lower due to considerable fixed costs. However, in our study we found the total annual council-wide cost to be higher than what was published by Borghi et al. (2015). Yet, detailed comparison with Borghi et al. was difficult because council level cost only included stewardship activities and it was unclear how dispensary and health centre costs were calculated given the number of health facilities in a council and the average annual health facility level cost. Consequently, cost to revenue ratios and cost per CHF member households were also higher than reported previously (Borghi et al., 2015).

In contrast to Borghi et al., we found in council A strong engagement of HFGC members in CHF mobilization activities, which reduced the burden of public health workers (Borghi et al., 2015). This showed the importance of considering council-specific CHF implementation practices and suggested that contrary to other places in Tanzania, HFGCs in council A were well informed about their roles and responsibilities (Kessy et al., 2008; Kessy, 2014). Additionally, it was argued before that cost resulting from mobilization activities could be reduced if all or most out-patients in public health facilities were covered by insurance (Borghi et al., 2015). However, we found that substantial mobilization activities would still be needed even if most out-patients had insurance coverage as seen in council A, where population coverage was just 11%, even though only 8% of out-patients were paying user fees. This demonstrated the relevance of taking into account the wider health financing context when looking at CHF implementation. Lastly, although our results undoubtedly confirm the lack of financial sustainability of the CHF observed by Borghi et al. (2015), they additionally showed that because the CHF was built into existing structures, there was considerable cross-subsidization in terms of financing sources paying for CHF administration (e.g. national tax-financed salaries, NHIF and user fee funds). This also meant that the CHF would be left with >70% of its revenues to purchase medicines and supplies and implement quality improvement activities at the health facility. It therefor again highlighted the importance of other health financing mechanisms in the analysis of CHF implementation.

### Way forward

In-line with what has been suggested by others, the results made clear that in order to make the CHF work, major improvements in CHF implementation practices would be indispensable (Stoermer et al., 2011, 2012; Mtei and Enemark, 2013; Kalolo et al., 2015, 2018; Ministry of Health and Social Welfare, 2015). Most importantly, our findings showed the importance of considering council-specific CHF implementation practices and the wider health financing context when looking at CHF performance. Changes in CHF implementation practices would need to go hand in hand with adaptions in other health financing policies (e.g. exemption, user fee, fund pooling policies) as the CHF cannot be looked at as a stand-alone system. It is highly questionable whether improvements in CHF implementation practices alone were feasible and scalable given the council-specific CHF premiums, CHF benefit packages, user fee policies and fund pooling mechanisms as well as when taking into account the exemption policies, other health financing mechanisms and healthcare-seeking behaviours. The question also remains whether such efforts to improve CHF implementation were value for money taking into account the already considerable contributions of other health financing mechanisms to CHF administration and the small contribution of the CHF to overall health financing.

Limited resources might potentially be better invested if in a first place the focus was on improving processes of major health financing sources coming from central level (Block Grants, Health Sector Basket Fund, Development Grants and MSD supply chain) in order to increase resource utilization and predictability of funding flows. This would lead more likely to a noticeable change in quality of care, because even little improvements in these processes could free up a substantial amount of money and human capacity. Improved quality might then in turn increase willingness of the community to contribute to health services as suggested by others (Bonu et al., 2003; World Health Organization, 2013; Adebayo et al., 2015). However, this would imply that for protecting the informal sector from financial hardship, they would need to be at least temporarily exempted from user fees until certain level of healthcare quality could be guaranteed. This could obviously not be done without increasing the level of funding for healthcare from central level through existing or new innovative financing solutions (Gilson and McIntyre, 2005; Dutta, 2015). Such changes may also have implications on several other parts of the system, including a potential increase in service utilization followed by a possible drop of quality of care (Gilson and McIntyre, 2005; Borghi et al., 2012; McIntyre et al., 2013; World Health Organization, 2013). However, given the problems with CHF implementation or CSIFs more generally, it could be worth considering conducting further research in this direction and advocate for the most pro-poor and cost-effective approach. In particular, a comprehensive study ought to be done, which compares the cost and other implications of abolishing user fees with the efforts required for effectively improving CHF implementation.

### Limitations of the study

Some data presented were collected from routine data and its documentation might have been erroneous. Yet, by verifying the numbers with additional sources available, it was assured to obtain data of reliable quality. Part of the analysis could only be done in council A, where detailed enough data were available. The lack of sufficient data in council B further supported the findings discussed above. For the cost calculations, also the cost of activities that would need to be done in the absence of the CHF was included. Though, these costs were apportioned according to the share of time spent on CHF administration. Additionally, it could be argued that the sample of informants providing costing information was too small to be representative for the council. However, most findings overlap well with what has been shown previously (Borghi et al., 2015). Finally, activities done by HFGCs were indirectly reported through the person responsible for CHF administration at the health facility. These estimates could thus be overestimated. Yet, even if the reported values were halved, apart from the absolute values for cost and time spent no statement reported in this study would change.

## Conclusion

Our results showed the importance of considering council-specific CHF implementation practices (overall CHF administration and the definition of the premium and benefit package) and the wider health financing context (council defined user fee policies and fund pooling mechanisms as well as exemption policies and other health financing mechanisms) when looking at CHF performance. Findings demonstrated that exemption policies and healthcare-seeking behaviour influenced negatively the maximum potential enrolment rate. Higher revenues from user fees, user fee policies and fund pooling mechanisms might have furthermore set incentives for care providers to prioritize user fees over CHF revenues. Bottlenecks in overall CHF administration diminished potential positive effects of a council level health insurance scheme and may ultimately have affected CHF enrolment. Costing results clearly pointed out the lack of financial sustainability of the CHF. The financial analysis however also showed that due to significant contributions from other financing mechanisms to CHF administration, the CHF could be left with >70% of its revenues for financing services.

Given the wider health financing context and healthcare-seeking behaviours, it is highly questionable whether improvements in CHF implementation practices alone were feasible and scalable. The question also certainly remains whether such efforts were value for money, and if limited resources were not better invested through primarily focusing on improving utilization and predictability of major health financing sources coming from central level. Therefore, this article calls for a realistic reconsideration of approaches taken to address the challenges in health financing and demonstrated that the CHF cannot be looked at as a stand-alone system.

## Supplementary Material

Supplementary Data
