# Supplementary material for: Looking at the bigger picture: how the wider health financing context affects the implementation of the Tanzanian Community Health Funds
Source: Health Policy Plan. 2019 Jan 25;34(1):12–23. doi: 10.1093/heapol/czy091 (PMC6479827; doi:10.1093/heapol/czy091)
Supplement: Supplementary Data [file czy091_supp.zip › czy091-Suppl_data/czy091_Supplementary_data.docx]

**Supplementary data**

**Annex 1**

Unit costs and source of financing for personnel were based on the national salary scales (Prime Minister’s Office Regional Administration and Local Government, 2013). Other unit costs and information about financing sources were derived from information given by respondents, CCHPs, annual combined TFPIRs, CHF specific documentation, other official documents collected and personal communication. Market prices were taken to value supplies (Table S2, supplementary data). Cost spanning multiple years were equally divided over the relevant time period. An exception was made for the CHF card, which was meant to last for five years but designed in a way that it could only be used for one year.

Costs for activities conducted by the ISAQH were excluded. Also, the cost for the overall process of formally exempting the poorest households from health service payments was not included. Furthermore, although routine CHMT supportive supervision was at least partially classified as a CHF administrative activity (Purchasing: Utilization reviews, quality assurance/monitoring) its cost was not taken into account here. Yet, this was discussed in detail elsewhere (Renggli et al., 2017). Start-up cost to introduce the CHF in a council and estimations of regional, zonal and national level cost were also not included.
