# Supplementary material for: Looking at the bigger picture: how the wider health financing context affects the implementation of the Tanzanian Community Health Funds
Source: Health Policy Plan. 2019 Jan 25;34(1):12–23. doi: 10.1093/heapol/czy091 (PMC6479827; doi:10.1093/heapol/czy091)
Supplement: Supplementary Data [file czy091_supp.zip › czy091-Suppl_data/czy091_Supplementary_data_table S1_.docx]

**Table S1** Personnel and time required for administrating the CHF in council A and B by type of activity.

|  | **Council A** | |  | **Council B** | |
| --- | --- | --- | --- | --- | --- |
|  | *Person involved (Quantity)* | *Time required (Number of informants)* |  | *Person involved (Quantity)* | *Time required (Number of informants)* |
| ***Mobilization*** |  |  |  |  |  |
| Individual sensitization during consultancy | D: HF i/c (1) HC&H: HF i/c (1/2) and other physicians (1/2) | D,HC&H: 0.9min/CHF member; 5.6min/user fee patient (N=8) |  | D: HF i/c (3/4) and other staff (1/4) HC: HF i/c (1/2) and other physicians (1/2) | D&HC: 0min/CHF member; 4.4min/user fee patient (N=7) |
| Group sensitization talks at the health facility | D: HF i/c (2/3) and other staff (1/3) HC&H: HF i/c (1/3), other physicians (1/3), other staff (1/3) | D,HC&H: 24.4min/week (N=8) |  | D: HF i/c (3/4) and other staff (1/4) HC: HF i/c (1/2) and other physicians (1/2) | D&HC: 39.7min/week (N=7) |
| Sensitization during the Village Assembly | D: Whole village attending, but VC (18), HF i/c (1), HFGC (5) included here | D: 5 out of 6 were invited 3.4 times a year: 8.5min on CHF out of 6.5h (N=6) |  | D: Whole village attending, but VC (28), HF i/c (1), HFGC (5) included here HC: Whole village attending, but VC (28), HFMT (1), HFGC (5) included here | D: 6 out of 6 were invited 3 times a year: 8.9min on CHF out of 3.75h (N=6) HC: 3 times a year: 8.9min on CHF out of 3.75h (N=7) |
| Sensitization out-reach activities (full day or on a daily base) | D: HFGC (5.2) HC: HFMT (3) H: CHF Co (1) C: CHF Co (1), NHIF (3), driver (1); CHF Co (1), CHW (94); CHMT (3 teams, 2 cars) (13.5) | D: 204 hours per year (N=6) HC: 1day/quarter (N=1) H: 1 day/year (N=1) C: 1 week/year (N=1); 2x1 day/year (N=2); 1x3 days/year (N=1) |  | D: HFGC (7) C: CHF Co (1), NHIF (3), driver (1); CHF Co (1) | D: 4 out of 6: 28 hours per year (N=6) C: 2 week/year (N=1); 1x6 day/year (N=1) |
| Sensitization of the Village Council | HC&H: VC (23), WDC (1) | HC&H: 15min on CHF out of 6h/quarter (N=4) for 4 village |  | D: HF i/c (1), VC (33) HC: VC (28), HFGC (5), HFMT (1) | D: 6 out of 6 were invited 2.9 times a year: 5.4min on CHF out of 4h (N=6) HC: 5.4min on CHF out of 4h/month (N=7) |
|  |  |  |  |  |  |
|  | **Council A** | |  | **Council B** | |
|  | *Person involved (Quantity)* | *Time required (Number of informants)* |  | *Person involved (Quantity)* | *Time required (Number of informants)* |
| Sensitization of the Ward Development Committee | ---------------------- | ---------------------- |  | D: HF i/c (1), WDC (31.5) HC: HF i/c (1), other physicians (1), WDC (30.5) | D: 5 out of 6 were invited 3.8 times a year: 8 min on CHF out of 4h (N=6) HC: 3.8 times a year 8 min on CHF out of 4h/quarter (N=6) |
| Registering, enrolling members; Billing, collecting contributions | D: HF i/c (1) HC&H: HF i/c (1/3), other physicians (1/3), other staff (1/3) | D,HC&H: 5.5min/new member (N=8) |  | D: HF i/c (2/3) and other staff (1/3) HC: Health staff | D&HC: 5.8min/new member (N=7) |
| ***Fund pooling*** |  |  |  |  |  |
| Deposing funds | D,HC&H:HF i/c (1) | D,HC&H: At council level (bank or CHF Co): 7.4h/month done with other activities (salary pick up --> 50%) for 9 months (in 3 months the accountant passes by) (N=7) |  | D: HF i/c (1) HC: HF i/c (1) | D: Into HF account: 16h/month (done with deposing user fee --> 33%) (N=6) HC: Using mobile payment system to deposit funds in the health facility account: 10min/month (N=1) |
| Collection of funds at health facilities | C: Accountant (1), driver (1) | C: 3x5.5 days/year (N=1) |  | ---------------------- | ---------------------- |
| Receipt of fund from health facilities | C: Accountant (1) | C: 5min/HF and month (9 months) (N=1) |  | ---------------------- | ---------------------- |
| Transferring funds to council account | C: Accountant (1) | C: 2x30min/week (N=1) |  | ---------------------- | ---------------------- |
| Applying for matching funds | C: CHF Co (1), accountant (2) | C: 39h/year (N=2) |  | C: CHF Co (1) | C: 1week/per month (N=1) |
| ***Stewardship*** |  |  |  |  |  |
| Training for CHF coordinator by NHIF | C: CHF Co (1) | C: 3-5 days/3 years (N=1) |  | ---------------------- | ---------------------- |
| Training for HFGC on tasks and duties | ---------------------- | ---------------------- |  | C: HFGC (182), HF i/c (31), CMO (1), CHMT (3), driver (1) | C: 11days/3year; 3.5h/HFGC; 25% on CHF (N=1) |
|  | **Council A** | |  | **Council B** | |
|  | *Person involved (Quantity)* | *Time required (Number of informants)* |  | *Person involved (Quantity)* | *Time required (Number of informants)* |
| Health Facility/Hospital Governing Committee meeting | D: HF i/c (1), HFGC (7) HC: HF i/c (2),HFGC (7) H: CHMT (5), HGC (6) | D&HC: 17.5min on CHF out of 3h/quarter (N=5) H: 36.25min on CHF out of 4.125h/quarter (N=2) |  | D: HF i/c (1), HFGC (7) HC: HF i/c (2),HFGC (7) | D&HC: 15.8 min on CHF out of 2.4h/quarter (N=7) |
| Village Council meeting | D: VC (23), HF i/c (1) | D: 3 out of 5 were invited every second month: 15min on CHF out of 6h (N=4) |  | ---------------------- | ---------------------- |
| Ward Development Council meeting | D,HC&H: HF i/c (1), WDC (24.4) | D: 3 out of 5 were invited HC&H: always invited 9.4 min on CHF out of 5.5h/quarter (N=4) |  | ---------------------- | ---------------------- |
| Health Facility/Hospital Management Team meetings | HC: HFMT (16.7) H: HMT (16.7) | HC&H: 12.5min on CHF out of 3.5h/month (N=3) |  | HC: HFMT (27) | HC: 20min on CHF out of 2h/month (N=1) |
| Council Health Service Board meeting | C: CHSB (7), CHMT or co-opted (4) | C: 3h on CHF out of 7.2h/quarter (N=3) |  | C: CHSB (7), CHMT or co-opted (4) | C: 3h total with about 22.5min on CHF/quarter (N=2) |
| Council Finance, Administration and Planning Committee meeting | C: CMO (1), CFAPC (24) | C: 1 day with about 2h on CHF/month (N=1) |  | ---------------------- | ---------------------- |
| Supply management | C: CHF Co (1) | C: 5h/month (N=1) |  | C: CHF Co (1) | C: 8h/week (N=1) |
| Data entry | C: CHF Co (1) | C: 4h/week (N=1) |  | ---------------------- | ---------------------- |
|  |  |  |  |  |  |
|  |  |  |  |  |  |
|  |  |  |  |  |  |
|  |  |  |  |  |  |
|  | **Council A** | |  | **Council B** | |
|  | *Person involved (Quantity)* | *Time required (Number of informants)* |  | *Person involved (Quantity)* | *Time required (Number of informants)* |
| Monthly reporting | D,HC&H: HF i/c (1) C: Accountant (1) | D: For 1/2 of dispensaries 7.4h/month for reports sent to council level together with other reports (--> 33%) and for 1/2 dispensaries reports are being picked up with others (N=6) HC&H: Fill NHIF form (90min/month); 7.4h (33%)/month reports sent to council level together with other reports (N=1) C: 2h/month (N=1) |  | D: HF i/c (1) HC: HF i/c (1) C: CHF Co (1); accountant (1) | D: 11.3h/month reports sent to council level together with other reports and money (-->33%) (N=6) HC: 8h/month reports sent to council level together with other reports (-->50%) (N=1) C: 1d/week (N=1); 3.5d/month (N=1) |
| Quarterly reporting | C: CHF Co (1) | C: 10h/quarter (N=1) |  | C: CHF Co (1) | C: 1d/quarter (N=1) |
| Yearly reporting | C: Accountant (1) | C: 4day/year (N=1) |  | C: CHF Co (1) | C: 1d/year (N=1) |
| Reporting during the CHMT meeting | C: CHF Co (1), CHMT (8 plus 3 co-opted) | C: 2x4h in total with 30min on CHF/year (N=1) |  | C: CHF Co (1), CHMT (8 plus 6 co-opted) | C: 2x1.75h total with 5min on CHF/month (N=1) |
| Budgeting | C: CMO (1) | C: 8h/week (N=1) |  | C: CMO (1) | C: 8h/week (N=1) |
| ***Purchasing*** |  |  |  |  |  |
| Utilization of funds, purchasing | C: CHF Co (1), CHMT/CMO (1) | C: 7h/week (N=2) |  | C: CHF Co (1), CMO (1) | C: 8h/week (N=2) |
| Supportive supervision | Done with NHIF advertising/marketing activities (see above); CHMT supportive supervision was excluded | ---------------------- |  | Done with NHIF advertising/marketing activities (see above); CHMT supportive supervision was excluded | ---------------------- |

Legend: C=Council; CFAPC=Council Finance, Administration and Planning Committee; CHF Co=CHF coordinator; CHMT=Council Health Management Team; CHSB=Council Health Service Board; CHW=Community Health Worker; CMO=Council Medical Officer; D=Dispensary; H=Hospital; HC=Health Centre; HF i/c = Health facility in-charge; HFGC=Health Facility Governing Committee; HFMT=Health Facility Management Team; VC= Village Council; WDC=Ward Development Committee
