# Supplementary material for: Looking at the bigger picture: how the wider health financing context affects the implementation of the Tanzanian Community Health Funds
Source: Health Policy Plan. 2019 Jan 25;34(1):12–23. doi: 10.1093/heapol/czy091 (PMC6479827; doi:10.1093/heapol/czy091)
Supplement: Supplementary Data [file czy091_supp.zip › czy091-Suppl_data/czy091_Supplementary_data_table S2.docx]

**Table S2** Unit cost of resources in Tanzanian Shillings (TSh).

| **Item** | **Unit cost** |
| --- | --- |
| Average salary of Council Medical Officer^1^ | 17'769/h° |
| Average salary of CHMT/CHSB/CFAPC^1^ | 9'332/h° |
| Average salary of CHF coordinator/NHIF personnel^1^ | 7'667/h° |
| Average salary of health facility in-charge (health centre)^1^ | 5’331/h° |
| Average salary of health facility in-charge (dispensary)/other physician (health centre)^1^ | 5’122/h° |
| Average salary of health accountant^1^ | 4'225/h |
| Average salary of HFGC member^1^ | 3'842/h° |
| Average salary of WDC/VC member^1^ | 3'338/h° |
| Average salary of medical personnel (e.g. nurse, medical attendant)^1^ | 3'012/h° |
| Average salary of community health worker^1^ | 1'761/h° |
| Average salary of driver^2^ | 2'596/h° |
| Per diem CHSB/HGC/CHF coordinator (council level)^3^ | 80'000/day |
| Per diem CHMT (village level)^3^ | 62'500/day |
| Per diem CHF coordinator/NHIF personnel/accountant (village level)^3^ | 60'000/day |
| Per diem driver (village level)^3^ | 50'000/day |
| Extra duty allowance for CHMT/HGC/CHSB/CHAPC^4^ | 31'250/day |
| Extra duty allowance for HFGC training (Council B)^5^ | 10'000/day&person |
| Extra duty allowance for out-reach activity of HFMT at health centre (Council A)^6^ | 20'000/day&person |
| Transport allowance CHF coordinator for NHIF training (Council A)^6^ | 20'000 return |
| Transport allowance for HGC/CHSB^5^ | 10'000 return |
| HFGC sitting allowance (Council A)^5^ | 10'000/meeting |
| HFGC sitting allowance (Council B)^5^ | 5'000/meeting |
| WDC/VC sitting allowance^6^ | 20'000/meeting |
| Transport to council (Council A)^6^ | 25'000 return |
| Transport to council for fund pooling (Council B)^6^ | 28'333 return |
| Transport to council for report submission from dispensary (Council B)^6^ | 26'833 return |
| Transport to council for report submission from health centre (Council B)^6^ | 30'000 return |
| Food and refreshment for HGC/NHIF training/CHSB/CFAPC^5^ | 10'000/day&person |
| CHF/accountant receipt book (50 pages)^6^ | 1'200/book |
| CHF card (lasts 1 year)^7^ | 1'400/card |
| NHIF register book (lasts for 5 years)^6^ | 10'000/book |
| Counter book for accounting at the health facility (lasts for 5 years) (Council B)^8^ | 3'000/book |
| Print out of page^8^ | 50/page |
| Diesel (1L per 7km)^8^ | 2'500/L |
| Training material for NHIF training^6^ | 2'000/person |
| Training material for HFGC training^5^ | 1'500/person |
| Rent for conference facility of NHIF training (for 50 people)^5^ | 100'000/day |

°Yearly salary was assumed to be equal to 52 weeks of 40 hours of work

^1^Source: Assumption based on information given by respondents and national salary scales (Prime Minister’s Office Regional Administration and Local Government, 2013)

^2^Source: Personal communication

^3^Salary and location-dependent; source: Information given by respondents, cross verified by official documentation collected by SR and IM

^4^Said to be half of the lowest per diem rate (village level); source: information and assumptions given by respondents, cross verified by personal communication

^5^Source: Information given by respondent, cross verified by CCHP budgets and quarterly combined TFPIRs collected by SR and IM

^6^Source: Information given by respondent

^7^Source: Information given by respondent, cross verified by CHF specific documentation from CHF coordinator and/or accountant collected by SR and IM

^8^Source: Market price collected by SR and IM
