# Supplementary figures and images for: Looking at the bigger picture: how the wider health financing context affects the implementation of the Tanzanian Community Health Funds
Source: Health Policy Plan. 2019 Jan 25;34(1):12–23. doi: 10.1093/heapol/czy091 (PMC6479827; doi:10.1093/heapol/czy091)

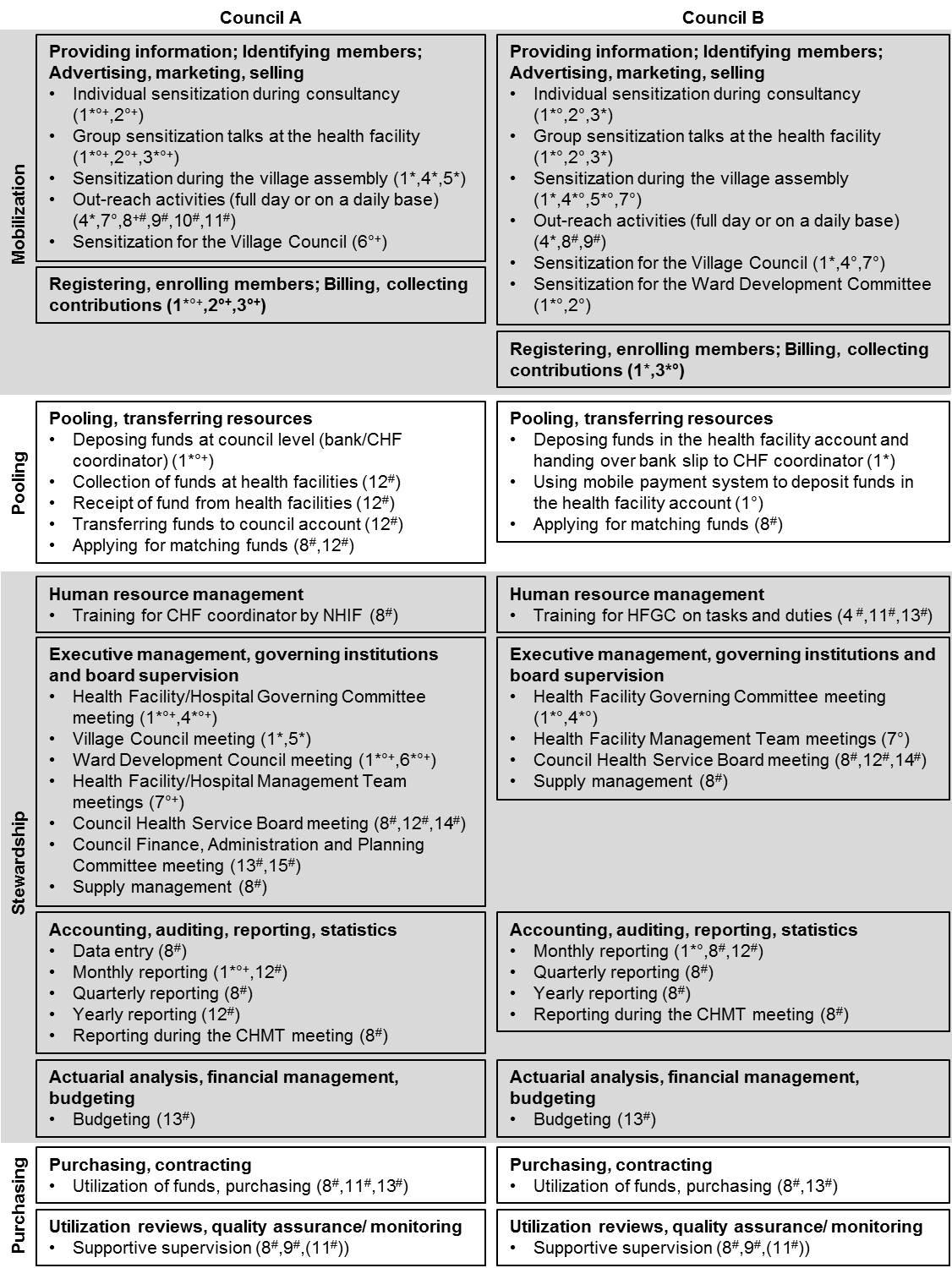

Supplement: Supplementary Data [file czy091_supp.zip › czy091-Suppl_data/czy091_Suppl_Figure_S1.jpg]
